# Improving incidence estimation in practice-based sentinel surveillance networks using spatial variation in general practitioner density

**DOI:** 10.1186/s12874-016-0260-x

**Published:** 2016-11-15

**Authors:** Cécile Souty, Pierre-Yves Boëlle

**Affiliations:** 1 Sorbonne Universités, UPMC Univ Paris 06, INSERM, Institut Pierre Louis d’épidémiologie et de Santé Publique (IPLESP UMRS 1136), F-75012 Paris, France; 2 Département de santé publique, AP-HP, Hôpital Saint-Antoine, F-75012 Paris, France

**Keywords:** Surveillance, Incidence estimation, General practitioners, Influenza-like illness, Surveillance networks, Epidemiological surveillance, GP density

## Abstract

**Background:**

In surveillance networks based on voluntary participation of health-care professionals, there is little choice regarding the selection of participants’ characteristics. External information about participants, for example local physician density, can help reduce bias in incidence estimates reported by the surveillance network.

**Methods:**

There is an inverse association between the number of reported influenza-like illness (ILI) cases and local general practitioners (GP) density. We formulated and compared estimates of ILI incidence using this relationship. To compare estimates, we simulated epidemics using a spatially explicit disease model and their observation by surveillance networks with different characteristics: random, maximum coverage, largest cities, etc.

**Results:**

In the French practice-based surveillance network – the “*Sentinelles*” network – GPs reported 3.6% (95% CI [3;4]) less ILI cases as local GP density increased by 1 GP per 10,000 inhabitants. Incidence estimates varied markedly depending on scenarios for participant selection in surveillance. Yet accounting for change in GP density for participants allowed reducing bias. Applied on data from the *Sentinelles* network, changes in overall incidence ranged between 1.6 and 9.9%.

**Conclusions:**

Local GP density is a simple measure that provides a way to reduce bias in estimating disease incidence in general practice. It can contribute to improving disease monitoring when it is not possible to choose the characteristics of participants.

**Electronic supplementary material:**

The online version of this article (doi:10.1186/s12874-016-0260-x) contains supplementary material, which is available to authorized users.

## Background

Surveillance networks for common acute conditions often rely on a sample of healthcare professionals who report disease cases seen in their practice [1, 2]. Various methods aim at optimizing the choice of these data providers and improving incidence estimates. For example, the locations of participating health professionals can be chosen to maximize population coverage [3, 4]. Another possibility is to make the monitored population representative in terms of age, sex or socio-economic status [5, 6]. It may also be possible to select participants so that estimates from the surveillance network are as close as possible to a reference epidemiologic signal, for example influenza-like hospitalizations when monitoring flu in the community [7].

Yet these *a priori* methods for selection implicitly assume that all health professionals or organizations would be willing to participate in surveillance upon proposal and not subject to turnover once recruited. These assumptions may indeed be reasonable in hospital or laboratory based surveillance networks, but not in surveillance networks based on general practitioners (GPs) in primary care. Indeed, in our experience, only a few percent of practicing GPs agree to participate in surveillance networks [8] and their participation is most of the times for a couple of years only [9].

More subtly, it has not been considered that physician density (physician per population ratio) changes the number and motives for medical consultations [10–12]: typically, in areas of low GP density, GPs see more cases of acute diseases because the population served is larger and a higher percentage of consultations is for treating acute diseases. On the contrary, in areas of high GP density, the number of acute disease cases seen by a GP is smaller, not so much as a result of less activity, but because fewer consultations are for treating acute diseases [13]. Accounting for local variation in physician density will be important when computing disease incidence indicators in health systems where patients are not registered to a unique GP practice, as is the case in most European countries [1, 14, 15].

To examine further the effects of GPs selection in primary care surveillance networks, we use a simulation approach and compare incidence estimates for various surveillance network structures similar to the French GP-based *Sentinelles* network [16]. Our goal is to explore how the spatial sampling of data providers affects the estimates of incidence and to propose improved estimators. We finally report an empirical comparison of incidence estimation based on influenza-like illness (ILI) in France.

## Methods

### The French *Sentinelles* network

The French general practitioners *Sentinelles* network [17] is a real-time epidemiologic surveillance system based on approximately 500 GPs, corresponding to 1% of all French GPs located all over the country [18]. Sentinel general practitioners (SGPs) are recruited and participate in surveillance on a voluntary basis. Differences between participating and non-participating GPs have been reported elsewhere [8, 19]. They report cases observed in their practice population, such as ILI cases, using a web interface or a dedicated software [20]. Incidences are calculated in real-time from their reports [19] allowing to detect outbreaks [21].

Here, we included the 301 SGPs participating to the French *Sentinelles* network during the 2012/13 influenza season. In addition to data provided by SGPs, we also obtained data on consultation volumes for all practicing French GPs and separately for SGPs from the national health insurance system [19, 22].

### Incidence estimation from cases reported by participating GPs

Incidence in the general population is computed from the number of patients with a specific combination of symptoms reported by the sample of participating SGPs [19, 23]. We present below estimators to compute incidence based on post-stratification. For most of the cases, incidence is first estimated by region (NUTS2 – Nomenclature of Territorial Units for Statistics level 2 [24]) and summed to estimate national incidence. Incidences estimates per population are computed as a ratio of estimated incidences divided by the area population (census data – National Institute of Statistics and Economics Studies [25]). A supplementary file contains all computational details (see Additional file 1).

### Horvitz-Thompson estimator: proportion of SGPs among GPs

A typical approach to reduce bias in non-representative samples is to use a Horvitz-Thompson estimator [26], where sampling weights correspond to the inverse of the inclusion probability. In the French *Sentinelles* network, these weights are computed as the percentage of SGPs participating in surveillance by region [19]. Indeed, the inclusion probability for SGP *k* is *π*_*k*_ = *nSGP*_*R*_/*nGP*_*R*_, where *nSGP*_*R*_ is the number of SGPs and *nGP*_*R*_ the total number of GPs in the region *R* where the SGP *k* practices. Thus, the national incidence estimator in period *t* is:

$$ {\widehat{I}}_{\pi }(t)={\sum}_k{\pi}_k^{-1}\cdot cases\left(k,t\right) $$

where *k* runs over participating SGPs and *cases(k,t)* is the number of cases reported by the SGP *k* during period *t*.

### Incidence estimator taking into account local GP density

All calculations are detailed in Additional file 1.

### Direct estimate using local GP density

Assume that the number of *cases* seen by a GP decreases with increasing local GP density *m* (number of GPs per population in the district or LAU1 - Local Administrative Units level 1 [24]) as *E(cases) = λ*/*m*, where *λ* is the incidence per population. The national incidence estimator is:

$$ {\widehat{I}}_{Dm}(t)={\sum}_k\frac{m_k}{m_{D,k}}\cdot {\pi}_k^{-1}\cdot cases\left(k,t\right) $$

where *m*_*D,k*_ *= nGP*_*D*_ *(k)/pop*_*D*_ *(k)* is the GP density at the departmental level (NUTS3 [24]) in SGP *k*’s department of practice.

### Calibrated estimate using local GP density

Calibration is a generic method to improve estimation when auxiliary information associated with measurements is available [27]. We use here the inverse of local GP density (LAU1) 1/*m* as auxiliary information for reported cases. The well-known ‘ratio-estimator’ [27] obtained with this approach has expression:

$$ {\widehat{I}}_{Cm}(t)={\sum}_k\frac{h_{R,k}}{m_{R,k}}\cdot {\pi}_k^{-1}\cdot cases\left(k,\ t\right) $$

where ${m}_{R,k}$ is the GP density in the region of practice of SGP *k* (=*nGP*_*R*_/*pop*_*R*_) and ${h}_{R,k}$ is the harmonic mean of GP densities among SGPs in the region *R* where SGP *k* practices, i.e. ${h}_{R,k}={nSGP}_R/\sum_{i\epsilon R}\left(1/{m}_i\right)$ where *i* runs over participating SGPs in *R*.

### Direct and calibrated estimates using local GP density and consultation volume

In a previous work [19], we found a positive association between the number of cases reported by SGPs and the number of consultations. Assuming joint proportionality of reported cases to GP density and consultation volume, a direct estimator of incidence is:

$$ {\widehat{I}}_{Dmc}(t)={\sum}_k\frac{m_k}{m_{D,k}}\cdot \frac{1}{\rho_{R,k}(t)}\cdot cases\left(j,\ t\right) $$

where *ρ*_*R,k*_ *(t)* = *cSGP*_*R*_ *(t)*/*cGP*_*R*_ *(t)* is the percentage of consultations by SGPs (*cSGP*_*R*_ *(t)*) among all consultations (*cGP*_*R*_ *(t)*) in region *R* where SGP *k* practices*,* during period *t.*

Using calibration proposed by Deville and Särndal [27], the incidence estimator calibrated on both local GP density and consultations is defined as follows:

$$ {\widehat{I}}_{Cmc}(t)={\displaystyle \sum_k\left({\pi}_k^{-1}\cdot cases\left(k,t\right)+{\left({\boldsymbol{t}}_x-{\widehat{\boldsymbol{t}}}_{x\pi}\right)}^{\prime}\cdot {T}^{-1}\cdot {\pi}_k^{-1}\cdot {{\boldsymbol{x}}_k}^{\prime}\cdot cases\left(k,t\right)\right)} $$

with $T=\sum_k{\pi}_k^{-1}{\boldsymbol{x}}_k{{\boldsymbol{x}}_k}^{\prime }$ where the auxiliary information ***x***_*k*_ = (1/*m*_*k*_, *ρ*_*R,k*_), ***t***_*x*_ is the population total of ***x***, and ${\widehat{\boldsymbol{t}}}_{x\pi }$ denote the Horvitz-Thompson estimator for the ***x***-vector.

### Investigating the relationship between GP density and reported cases by SGPs

We analysed the number of cases reported to the *Sentinelles* network during an epidemic by a SGP using Poisson regression with dependant variable the local GP density (district level – LAU1). We used the data over the last five seasons (2010/11 to 2014/15) for each SGP.

### Influenza epidemics simulations

Influenza epidemics were simulated using a spatially explicit age-structured model including population and commuting data in France [16]. Simulations were performed at the district level (LAU1 - 3708 districts in France) and yielded weekly incidence data. Influenza cases in the 98 districts with no GPs were allocated to the neighbouring districts.

In each district, the average number of cases seen by a GP in 1 week was computed as the ratio of number of new cases in the district divided by the number of GPs in that district. We assumed that the actual number of cases reported by a GP had a Poisson distribution about this average.

### Evaluation of sample weights for incidence estimation

#### Simulated GPs networks

To investigate the impact of geographical location of GPs participating to surveillance, we simulated networks by choosing data providers from the 60,000 practicing French GPs. Network sizes was limited to 300 to be similar to the existing French *Sentinelles* network.

We first investigated 1000 “*random networks*” including 300 GPs chosen at random from all GPs. Next, we selected a network including one GP taken in each of the districts of the *administrative centres* at the NUTS3 level (*n* = 96 departments in France). We also selected *low GP density* network*,* with GPs from the three districts with the lowest GP density in each department, and *high GP density* network where GPs were taken from the three districts with the highest GP density. Finally, we used the maximum coverage algorithm proposed by Polgreen et al. [3] to maximize the population covered by participating GPs using 300 GPs and a distance of 14 kilometres (8.7 miles – the average diameter of a district).

#### Comparing estimated incidence to real incidence

To compare incidence estimates based on the various surveillance networks, we computed the weekly average relative difference in percent and the root mean squared error (RMSE) between estimated and simulated incidence. Estimates were computed using the Horvitz-Thompson estimator *Î*_*π*_ and the estimators taking into account local GP density *Î*_*Dm*_ *and Î*_*Cm*_. To account for variability in number of reported cases by participating GPs (Poisson distribution), we replicated estimations 100 times and reported the average.

#### Empirical assessment: subsampling SGPs in the Sentinelles network

To investigate empirically the characteristics of the estimators on real data, we used a subsampling approach. We estimated incidence using 75% of all participating SGPs only, and varied randomly the 75% selected. We computed the three estimators *Î*_*π*_ *, Î*_*Dm*_ *and Î*_*Cm*_ as defined above (Horvitz-Thompson and both defined with local GP density) during the 2012/13 influenza epidemic (2012 week 51^th^ to 2013 week 11^th^). Using results from subsampling, we estimated the standard deviation of estimated incidence and the coefficient of variation.

Finally, we applied all estimators to estimate ILI incidence over the 5 years period from 2009 week 32^th^ to 2014 week 31^th^. Comparisons between estimators were done during epidemic periods as defined by the *Sentinelles* network [17, 21].

## Results

### Spatial distribution of GPs according to population in France

**Fig. 1:**
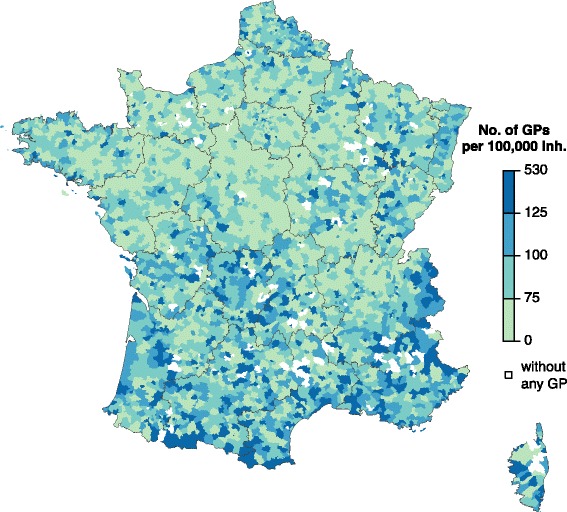
General practitioners (GP) density by district (LAU1) in France (number of GPs per 100,000 inhabitants) in 2012. *Dark grey lines* represented boundaries of the 22 French regions (NUTS2)

Overall, the average GP density in France was 96 GPs per 100,000 inhabitants, ranging from 12 to 526 GP per 100,000 inhabitants (except in 98 districts (2.6%) without a GP) (Fig. 1). The 301 SGPs of the *Sentinelles* network came from 260 districts all over the country. In the districts of practice of the SGPs, the average GP density was slightly lower than in overall France (94 GPs per 100,000 inhabitants).

### Number of cases per SGP and GP density using data from the French *Sentinelles* network

**Fig. 2:**
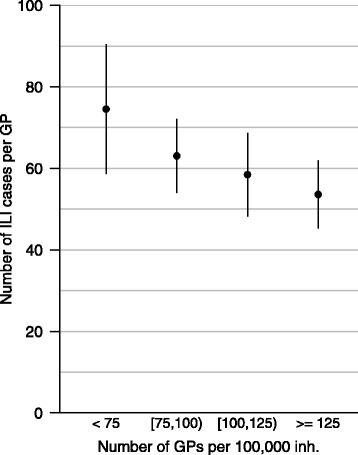
Average cumulative numbers of influenza-like illness (ILI) cases reported by French sentinel general practitioners during the 2012/13 influenza epidemic versus general practitioners (GP) density (number of GPs per 100,000 inhabitants) by district (LAU1), France

SGPs practicing in places with high GP density, reported fewer ILI cases over the duration of an epidemic (Fig. 2). On average, the number of cases was reduced by 3.6% (95% CI [3; 4]) as GP density increased by 10 GP per 100,000 inhabitants (*p* < 2.10^−16^). In other words, during a typical flu epidemic, a GP practicing in a district with 75 GP/100,000 inhabitants (first quartile of GP density in SGPs) reported 42 cases while a GP practicing in a district with 125 GP/100,000 (third quartile of GP density in SGPs) reported only 34.

### Incidence estimates using simulated GPs networks

**Fig. 3:**
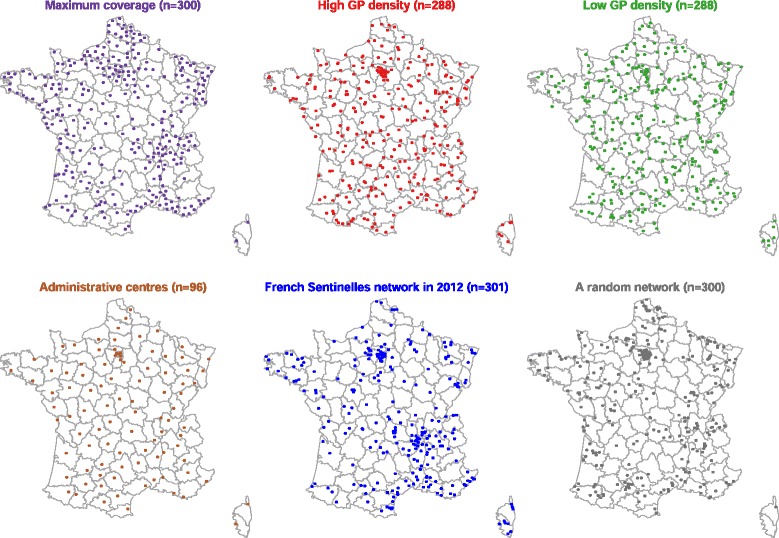
Spatial spread of general practitioners (GPs) involved in the simulated practice-based surveillance networks, France; number of GPs involved in each network is reported in brackets

The geographical location of GPs included in the simulated surveillance networks did not show specific spatial patterns (Fig. 3) except for fewer GPs included in the *administrative centres* network (96 instead of 300). Visually, the actual *Sentinelles* network looked patchier than the *maximum coverage*, *high/low GP density* and *administrative centres* networks but similar to *random networks*.

**Fig. 4:**
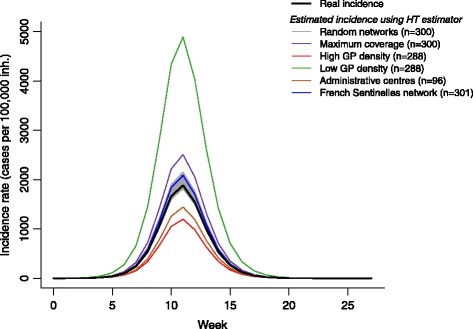
Influenza-like illness incidence rates from simulations (real) and estimated by Horvitz-Thompson estimator using various general practitioners (GPs) networks in France. Number of GPs involved in each network was reported in brackets

For the same simulated epidemic, estimated incidence was highly dependent on data provider selection using the Horvitz-Thompson estimator *Î*_*π*_ (Fig. 4). In *random networks,* incidence was estimated almost without bias (+4%; RMSE = 59 cases per 100,000 inhabitants) but with substantial variation from one network to the next. With the *administrative centres* network, incidence was underestimated by an average of 24% (RMSE = 306). The largest underestimates were obtained for the *high GP density* network, where incidence was one third less than the actual values (−37%; RMSE = 467). Conversely, the *low GP density* network led to the largest overestimates, with estimated incidence more than one and half the actual incidence (+164%; RMSE = 2370). The maximal coverage network, while covering 79% of the French population with 300 GPs, overestimated incidence by one third (+33%; RMSE = 425). Finally, in the network corresponding to the actual *Sentinelles* network, incidences estimates were on average 10% higher than expected (RMSE = 133).

#### Introducing local GP density in incidence estimators

**Fig. 5:**
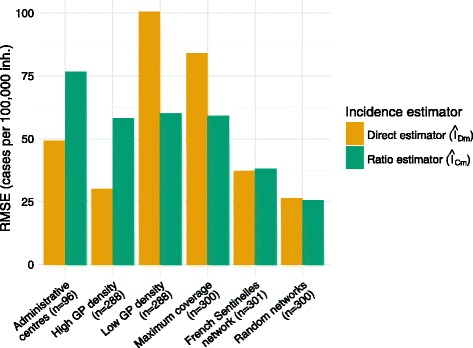
Root mean square error of incidence estimates based on estimators accounting for local GP density compared to real incidences in the various simulated networks

Taking into account local GP density allowed substantial bias reduction in all simulated networks: estimates were always closer to the actual incidence by comparison with the Horvitz-Thompson estimator. In random networks, the ratio estimator *Î*_*Cm*_ and the direct estimator *Î*_*Dm*_ performed similarly (RMSE = 26). For other network choices, the situations were contrasted: the ratio estimator *Î*_*Cm*_ performed better for *low GP density* and *maximum coverage* networks and the direct estimator *Î*_*Dm*_ for *administrative centres* and *high GP density* networks (Fig. 5).

### Subsampling SGPs from the French *Sentinelles* network

We next compared estimators *Î*_*π*_, *Î*_*Dm*_ and *Î*_*Cm*_ on the *Sentinelles* data for 2012/13 influenza epidemic. The average standard deviation decreased from 24 cases per 100,000 inhabitants for *Î*_*π*_ to 19 for *Î*_*Dm*_ and *Î*_*Cm*_. Likewise, the coefficients of variation were 5.2% when using *Î*_*Dm*_ and 5.3% with *Î*_*Cm*_, less than with *Î*_*π*_ (5.9%).

In general, modifications of the original *Î*_*π*_ led to lower incidences estimates, as expected given the average GP density was slightly lower for the *Sentinelles* network than for overall France. Using the Horvitz-Thompson estimator as a reference with whole SGPs’ data, the estimated incidence was on average 1.6% less for the direct estimator *Î*_*Dm*_, 8.4% less for the ratio estimator *Î*_*Cm.*_ Finally, calibration on both local GP density and consultation volume led to similar performance compared with calibration on local GP density alone, with a reduction by 9.9% using *Î*_*Cmc*_ and by 4.7% using *Î*_*Dmc*_.

## Discussion

Reducing bias in disease incidence estimates from GP surveillance networks is a challenge when participants are volunteers rather than carefully chosen by administrators. Here, we showed that bias due to variations in local GP density around participants could be reduced using weighted estimators, and supported this conclusion with empirical evidence. This approach could be used to introduce more flexibility for developing algorithms to identify the best placement of data providers [3–7].

We found that local GP density changed the reports of GPs to surveillance systems, with less ILI cases reported as local GP density increased. This was also true with acute diarrhea and chickenpox, two other diseases monitored by the *Sentinelles* network. For example, the number of acute diarrhea cases reported by SGPs during the epidemic was reduced by 3.4% (95% CI [2.9;3.9] (*p* < 0.0001) and the number of yearly chickenpox cases was reduced by 6.4% (95% CI [5.9;6.8]) (*p* < 0.0001) when the GP density increase by 10 GP per 100,000 inhabitants. There is indeed a known effect of GP density on their activity which reflects competition between physicians [11]. While the number of consultations may be slightly reduced with increasing GP density, it is mostly the nature of consultations that changes [13]. More consultations are for prevention, or follow-up of chronic diseases in high GP density areas, leading to fewer acute disease cases per GP and possible bias in incidence estimation.

Surveillance networks in general practice must satisfy a number of constraints: sensitivity to change in incidence; large spatial coverage; robustness regarding dynamic changes in the network composition; and efficient use of resources. In this last respect, seemingly reasonable solutions may have undesirable features: we found here that selecting one GP in each of the 100 largest French towns would lead to large underestimation of incidence. Several approaches have been described to the optimal selection of data providers in surveillance, taking into account spatial coverage or representativeness of monitored population [3–7]. In our experience, the effective use of such approaches in GP based surveillance is difficult as it is not possible to have a stable roster of GPs who want to participate in surveillance [8]. The interest of *a priori* computations to identify an optimal set of providers is further reduced if candidates can refuse participation. Furthermore, turnover of participants, which is common [1], may jeopardize carefully arranged providers sets. Interestingly, we found that in France, the maximum coverage algorithm proposed by Polgreen et al. [3] ended up selecting places with slightly below average GP density, leading to upward biased incidences. It may be possible to alter this coverage algorithm so that the only GPs proposed for inclusion are those practicing in areas with average GP density, at the price of reducing coverage. Unexpectedly, the self-selection of GPs in the *Sentinelles* network led to an average GP density in the monitored regions that was close to the average national GP density. This suggests that participation to surveillance is indeed independent from local GP density, a feature that is required in the estimators described above.

When *a priori* optimal selection of data providers for surveillance is not carried out, *post-hoc* stratification can address the issue. This requires identifying characteristics associated with the estimated quantity, as local GP density with the number of cases reported by SGPs. At worst, calibration with an irrelevant characteristic increases variance but not bias [28]. A feature of practical importance is that local GP density is in most situations easy to obtain, when other candidate characteristics to reduce bias, like consultation numbers [19], is more difficult to obtain. Additionally, GP density is easily recomputed when a GP joins or stops surveillance. Post-stratification may furthermore be used in all kinds of GP selection schemes, including the algorithmic approaches described above.

We used a simulation approach to investigate the impact of spatial sampling of providers on incidence estimates. The model for ILI simulations [16] was previously shown to reproduce the major characteristics of flu epidemics. The various observation networks that we compared corresponded with plausible choices, for example large cities (similar to the 122 cities mortality system of CDC [29]) or maximum coverage. That there is bias associated with these choices is important to stress, as these may be considered in setting up surveillance networks. As with all surveillance network data, empirical validation is difficult as the real incidence is unknown. The performance of estimators is often assessed by bias^2^ + variance. Since improved estimates do not increase bias, reducing variance provides good evidence of improved performance in our situation.

## Conclusions

Bias in incidence estimated through epidemiological data from voluntary surveillance networks could be reduced by using sampling weights based on local GP density. It can contribute to improving disease monitoring when the selection of participants is not controllable.

## Additional file

Additional file 1: “Computational details”. Detailed computations of incidence estimators. (DOCX 39 kb)

## Abbreviations

CI: Confidence interval
GP: General practitioner
ILI: Influenza-like illness
LAU: Local administrative units
NUTS: Nomenclature of territorial units for statistics
RMSE: Root mean squared error
SGP: Sentinel general practitioner

## References

[1] Deckers JGM, Paget WJ, Schellevis FG, Fleming DM (2006). European primary care surveillance networks: their structure and operation. Fam Pract.

[2] Schlaud M, Brenner MH, Hoopmann M, Schwartz FW (1998). Approaches to the denominator in practice-based epidemiology: a critical overview. J Epidemiol Community Health.

[3] Polgreen PM, Chen Z, Segre AM, Harris ML, Pentella MA, Rushton G (2009). Optimizing influenza sentinel surveillance at the state level. Am J Epidemiol.

[4] Fairchild G, Polgreen PM, Foster E, Rushton G, Segre AM (2013). How many suffice? A computational framework for sizing sentinel surveillance networks. Int J Health Geogr.

[5] Pérez-Farinós N, Galán I, Ordobás M, Zorrilla B, Cantero JL, Ramírez R (2009). A sampling design for a sentinel general practitioner network. Gac Sanit.

[6] Byass P (2003). Empirical modelling of population sampling: lessons for designing sentinel surveillance. Public Health.

[7] Scarpino SV, Dimitrov NB, Meyers LA (2012). Optimizing provider recruitment for influenza surveillance networks. PLoS Comput Biol.

[8] Chauvin P, Valleron A-J (1995). Attitude of French general practitioners to the public health surveillance of communicable diseases. Int J Epidemiol.

[9] Chauvin P, Valleron A-J (1998). Participation of French general practitioners in public health surveillance: a multidisciplinary approach. J Epidemiol Community Health.

[10] Béjean S, Peyron C, Urbinelli R (2007). Variations in activity and practice patterns: a French study for GPs. Eur J Health Econ.

[11] Léonard C, Stordeur S, Roberfroid D (2009). Association between physician density and health care consumption: a systematic review of the evidence. Health Policy.

[12] Dumontet M, Franc C (2015). Gender differences in French GPs’ activity: the contribution of quantile regressions. Eur J Health Econ.

[13] Delattre E, Dormont B (2003). Fixed fees and physician-induced demand: a panel data study on French physicians. Health Econ.

[14] Schlaud M. Comparison and Harmonisation of Denominator Data for Primary Health Care Research in Countries of the European Community: The European Denominator Project. IOS Press; 1999.

[15] Fleming DM (1998). The role of research networks in primary care: based on a presentation at WONCA Dublin in June 1998. Eur J Gen Pract.

[16] Charaudeau S, Pakdaman K, Boëlle P-Y (2014). Commuter mobility and the spread of infectious diseases: application to influenza in France. PLoS One.

[17] French general practitioners Sentinelles network. http://www.sentiweb.fr/?lang=en. Accessed 2 May 2016.

[18] Flahault A, Blanchon T, Dorléans Y, Toubiana L, Vibert JF, Valleron AJ (2006). Virtual surveillance of communicable diseases: a 20-year experience in France. Stat Methods Med Res.

[19] Souty C, Turbelin C, Blanchon T, Hanslik T, Le Strat Y, Boëlle P-Y (2014). Improving disease incidence estimates in primary care surveillance systems. Popul Health Metr.

[20] Turbelin C, Boëlle P-Y (2010). Improving general practice based epidemiologic surveillance using desktop clients: the French sentinel network experience. Stud Health Technol Inform.

[21] Costagliola D, Flahault A, Galinec D, Garnerin P, Menares J, Valleron AJ (1991). A routine tool for detection and assessment of epidemics of influenza-like syndromes in France. Am J Public Health.

[22] Tuppin P, de Roquefeuil L, Weill A, Ricordeau P, Merlière Y (2010). French national health insurance information system and the permanent beneficiaries sample. Rev Epidemiol Sante Publique.

[23] Turbelin C, Souty C, Pelat C, Hanslik T, Sarazin M, Blanchon T (2013). Age distribution of influenza like illness cases during post-pandemic A(H3N2): comparison with the twelve previous seasons, in France. PLoS One.

[24] Eurostat (European Commission). NUTS - Nomenclature of territorial units for statistics. http://ec.europa.eu/eurostat/web/nuts/overview. Accessed 2 May 2016.

[25] National Institute of Statistics and Economics Studies - France. http://www.insee.fr/en/. Accessed 2 May 2016.

[26] Horvitz DG, Thompson DJ (1952). A generalization of sampling without replacement from a finite universe. JASA.

[27] Deville J-C, Särndal C-E (1992). Calibration estimators in survey sampling. JASA.

[28] Miratrix LW, Sekhon JS, Yu B (2013). Adjusting treatment effect estimates by post-stratification in randomized experiments. J R Stat Soc Series B Stat Methodol.

[29] Baron RC, Dicker RC, Bussell KE, Herndon JL (1988). Assessing trends in mortality in 121 U.S. cities, 1970–79, from all causes and from pneumonia and influenza. Public Health Rep.

