# Supplementary material for: Improving incidence estimation in practice-based sentinel surveillance networks using spatial variation in general practitioner density
Source: BMC Med Res Methodol. 2016 Nov 15;16:156. doi: 10.1186/s12874-016-0260-x (PMC5111194; doi:10.1186/s12874-016-0260-x)
Supplement: Additional file 1: — “Computational details”. Detailed computations of incidence estimators. (DOCX 39 kb) [file 12874_2016_260_MOESM1_ESM.docx]

# Supplemental methods: computational details

We presented here the derivation of incidence estimators used in the article, implementing direct approaches and calibration. For most of the cases, incidence is first estimated by region (NUTS2 [1]) and summed to estimate national incidence.

The Horvitz-Thompson (HT) estimator [2] of incidence is computed with sampling weights for each sentinel general practitioner (SGP) *k* corresponding to the inverse of its inclusion probability (*π_k_*) in the sample, computed as the proportion of general practitioners (GPs) participating in surveillance in a region : *π_k_=nSGP_R_/nGP_R_*. At the national level, the HT incidence estimator for period *t* is:

$$\hat{I}_{\pi}(t)=\sum_{k} \pi_{k}^{-1}\cdot cases(k,t)$$

where k runs over participating SGPs and *cases(k,t)* is the number of cases reported by the SGP *k* during period *t*.

## Incidence estimator taking into account local GP density

### Incidence estimator based on a direct approach

An underlying assumption to the derivation of HT sampling weights is that all GPs in a region see the same number of cases on average. Here, we adopt the assumption that the per population incidence in period *t*, denoted *λ_Dm_(t)*, is constant at the NUTS3 level (department level), but that the number of cases seen by a SGP during this period is inversely proportional to the GP density in the district (LAU1) of practice denoted *m*, i.e. *E(cases(k,t)) = λ_Dm_(t)/m*, with *m = nGP/pop* where *nGP* and *pop* are the number of GPs and population in the district. An estimate of *λ_Dm_(t)* can be formed as a weighted mean of individual SGP reports as $\hat{\lambda}_{Dm}\left( t \right)=\sum_{k\in D} \alpha_{k}\cdot m_{k}\cdot cases(k,t)$ with $\sum_{k\in D} \alpha_{k}=1$

A simple choice for *α_k_* is to give the same weight to each SGP, i.e. *α_k_* = 1/*nSGP_D_*, yielding $\hat{\lambda}_{Dm}\left( t \right)=1/{{nSGP}_{D}}\cdot\sum_{k\in D} m_{k}\cdot cases\left( k,t \right)$, where *nSGP_D_* is the number of SGP at the department level. In this case, we recover national incidence as:

$$\hat{I}_{Dm}\left( t \right)= \sum_{k} \frac{m_{k}}{m_{D,k}}\cdot\pi_{k}^{-1}\cdot cases\left( k,t \right)$$

with *m_D,k_ = nGP_D_(k)/pop_D_(k)* is the GP density in the department of practice of SGP *k*. As a simplification, *nSGP_D_* has been replaced by *nGP_D_*nSGP_R_/nGP_R_* = *nGP_D_*π_k_* assuming that the percentage of SGPs is the same in all departments.

If a variance model is assumed for *cases*, inverse variance weighting leads to a choice of *α_k_* yielding the least variable estimator. For example, assuming *cases(k,t)* ~ Poisson(*λ_Dm_(t) / m_k_*), leads to *α_k_* # 1/*m_k_*, and $\hat{\lambda}_{Dm}\left( t \right)=\sum_{k\in D} cases\left( k,t \right)/\sum_{k\in D} {(1}/{m_{k})}$. This in turn yields the so-called ‘ratio-estimator’ that is described below.

### Incidence estimator using calibration

If auxiliary information *x,* correlated with observed information *y*, is available for each unit in the population, calibration of ordinary sampling weights (from HT estimator) may improve the precision of estimates. We applied the general calibration estimator as described by Deville and Särndal [3]: the calibrated weights *w_k_* are as close as possible to the ordinary HT sampling weights *π_k_^-1^* using the chi-squared distance, subject to the calibration constraints.

Assuming *x_k_* the value of the auxiliary variable *x* for the SGP *k* is one-dimensional, the calibration estimator of the total number of disease cases in region *R* is:

$$\hat{I}_{Cx}(R,t)=\sum_{k\in R} w_{k}\cdot cases(k,t)=\sum_{k\in R} \pi_{k}^{-1}\left( 1+\frac{t_{x,R}-t_{x\pi,R}}{\sum_{i\epsilon R} \pi_{i}^{-1}q_{i}x_{i}^{2}}q_{k}x_{k} \right)\cdot cases(k,t)$$

where *i* runs over SGPs practicing in *R*, *q_k_* is an arbitrary weight for SGP *k*, *t_x,R_* is the known population total of *x* in the region *R* and *t_xπ,R_* the HT estimator for x in the region *R*.

Here, the auxiliary information available for each GP in the population is the GP catchment population size, corresponding to the inverse of local GP density, assumed to be correlated with the number of cases reported by SGPs. The total of this auxiliary variable in the region *R* is *t_x,R_* = *pop_R_*.

Choosing a uniform weighting 1/*q_k_*=1 leads to the national incidence estimator *Î_Cm_unif_(t)* :

$$\hat{I}_{Cm\_unif}(t)=\sum_{R} \sum_{k\epsilon R} \left( \pi_{k}^{-1}+\frac{{pop}_{R}-\pi_{k}^{-1}\cdot\sum_{i\epsilon R} 1/m_{i}}{\sum_{i\epsilon R} {1/m_{i}}^{2}}\cdot\frac{1}{m_{k}} \right)\cdot cases(k,t)$$

Using weights *q_k_* = 1/*x_k_* = *m_k_* yields the so-called ‘ratio estimator’ [3] with expression:

$$\hat{I}_{Cm}(t)=\sum_{k} \frac{h_{R,k}}{m_{R,k}}{\cdot\pi}_{k}^{-1}\cdot cases\left( k, t \right)$$

where *m_R,k_* is the GP density in the region of practice of SGP *k* (=*nGP_R_*/*pop_R_*) and *h_R,k_* is the harmonic mean of GP densities among SGPs in the region R where SGP *k* practices, i.e. $h_{R,k}= {nSGP}_{R}/\sum_{i\in R} (1/m_{i})$ where *i* runs over participating SGPs in *R*.

## Incidence estimator taking into account local GP density and number of GP consultations

### Incidence estimator based on a direct approach

In a previous work [4], we showed that number of cases reported by SGPs was positively associated with the number of consultations. To account for both variations due to consultations and GP density, we first derive directly an incidence estimator under the hypothesis
E(*cases(k,t)*) = *λ_Dmc_(t) / m_k_* * *c_k_(t)*, where *c_k_(t)* is the number of consultations for SGP *k* during period *t*.

As above, it is easy to derive an estimator for the per population incidence at the department level: $\hat{\lambda}_{Dmc}\left( t \right)={(1}/{{nSGP}_{D}}{\cdot{cGP}_{D}(t)}/{{nGP}_{D})}\cdot\sum_{k\in D} m_{k}/c_{k}(t)\cdot cases\left( k,t \right)$

In our application, number of consultations by GPs is aggregated at the regional (NUTS2) level. We therefore replaced *c_k_(t)* by *cSGP_R_(t)*/*nSGP_R,_* the mean number of consultations per SGP in *R*. Likewise, we replace *cGP_D_(t)*/*nGP_D_* by *cGP_R_(t)*/*nGP_R_*. Moreover, as above *nSGP_D_* was replaced by *nGP_D_* _*_ *π_k_*_._ The consultation and GP density adjusted direct estimator is therefore:

$$\hat{I}_{Dmc}\left( t \right)= \sum_{k} \frac{m_{k}}{m_{D,k}}\cdot\frac{1}{\rho_{R,k}(t)}\cdot cases(j,t)$$

where *ρ_R,k_(t)* = *cSGP_R_(t)*/*cGP_R_(t)* is the percentage of consultations by SGPs among all consultations in region *R,* during period *t*.

### Incidence estimator 2-dimensional ranking

The generalized estimator of Deville and Särndal [3] can also easily be adapted to multidimensional auxiliary information, here ***x****_k_* = [1/*m_k_,* *ρ_R,k_(t)*]. Using the chi-squared distance and arbitrary uniform weights *q_k_*=1, we obtained a dual calibrated incidence estimator:

$$\hat{I}_{Cmc}(t)=\sum_{k} \left( \pi_{k}^{-1}\cdot cases\left( k,t \right)+\left( \boldsymbol{t}_{x}-{\hat{\boldsymbol{t}}}_{x\pi} \right)^{'}\cdot T^{-1}\cdot\pi_{k}^{-1}\cdot{\boldsymbol{x}_{k}}^{'}\cdot cases(k,t) \right)$$

where *t_x_* is the total of ***x*** in the population, ***t****_xπ_* denote the HT estimator for the x-vector with expression, $\boldsymbol{t}_{x\pi} = \left[ \begin{matrix} \sum_{k} \pi_{k}^{-1}\cdot\frac{1}{m_{k}} \\ \sum_{k} \pi_{k}^{-1}\cdot\rho_{R,k}(t) \end{matrix} \right]$ and $T =\left[ \begin{matrix} \sum_{k} \pi_{k}^{-1}\cdot\frac{1}{m_{k}^{2}} & \sum_{k} \pi_{k}^{-1}\cdot\frac{1}{m_{k}}{\cdot\rho}_{R,k}(t) \\ \sum_{k} \pi_{k}^{-1}\cdot\frac{1}{m_{k}}\cdot\rho_{R,k}(t) & \sum_{k} \pi_{k}^{-1}\cdot{\rho_{R,k}(t)}^{2} \end{matrix} \right]$, assuming that the inverse of *T* exists.

## References

1. Eurostat (European Commission). NUTS - Nomenclature of territorial units for statistics. http://ec.europa.eu/eurostat/web/nuts/overview. Accessed 2 May 2016.

2. Horvitz DG, Thompson DJ. A Generalization of Sampling Without Replacement From a Finite Universe. JASA. 1952;47:663–85.

3. Deville J-C, Särndal C-E. Calibration Estimators in Survey Sampling. JASA. 1992;87:376–82.

4. Souty C, Turbelin C, Blanchon T, Hanslik T, Le Strat Y, Boëlle P-Y. Improving disease incidence estimates in primary care surveillance systems. Popul Health Metr. 2014;12:19.
